# Single anastomosis duodenal-ileal bypass with sleeve gastrectomy (SADI-S): experience from a high-bariatric volume center

**DOI:** 10.1007/s00423-022-02501-z

**Published:** 2022-03-29

**Authors:** Francesco Pennestrì, Luca Sessa, Francesca Prioli, Giulia Salvi, Pierpaolo Gallucci, Luigi Ciccoritti, Francesco Greco, Carmela De Crea, Marco Raffaelli

**Affiliations:** 1 U.O.C. Chirurgia Endocrina E Metabolica, Centro Dipartimentale Di Chirurgia Endocrina E Dell’Obesità, Fondazione Policlinico Universitario Agostino Gemelli IRCCS, Rome, Italy; 2 Università Cattolica del Sacro Cuore, Rome, Italy; 3 Centro Malattie Endocrine E Obesità, Fondazione Gemelli Giglio Cefalù, Cefalù (Palermo), L.go A. Gemelli 8, 00168 Rome, Italy

**Keywords:** SADI-S, SADI, Duodenal switch, Sleeve gastrectomy, Post-operative complications, Bariatric outcomes

## Abstract

**Purpose:**

Biliopancreatic diversion with duodenal switch and single anastomosis duodenal-ileal bypass with sleeve gastrectomy (SADI-S) are technically demanding hypo-absorptive bariatric procedures generally indicated in super-obese patients (BMI ≥ 50 kg/m^2^). Data from the literature prove the procedure to be safe and effective, with promising bariatric and metabolic effects. Anyway, international societies support the creation of multicentric national and international registries to obtain more homogeneous data over the long period. We aimed to report our experience with this procedure.

**Methods:**

Among 2313 patients who underwent bariatric procedures at our institution, between July 2016 and August 2021, 121 (5.2%) consenting patients were scheduled for SADI-S as primary (SADIS) or revisional procedure after sleeve gastrectomy (SADI) (respectively 87 and 34 patients). Early and late post-operative complications, operative time, post-operative stay, and follow-up data were analyzed.

**Results:**

Overall, the median preoperative BMI was 52.3 (48.75–57.05) kg/m^2^ with a median age of 44 (39–51) years, the median operative time was 120 (100–155) min. Complications at 30th-day post-op were registered in 4 (3.3%) patients and late complications in 4 (3.3%) patients. At a median follow-up of 31 (14–39) months, the median percentage excess weight loss was 79.8 (55.15–91.45)%, and the median total weight loss was 57.0650 (43.3925–71.3475)%.

**Conclusion:**

Our data, coherently with the literature, confirm that SADI-S is a safe, effective procedure with acceptable complications rate. Larger studies with longer follow-ups are necessary to draw definitive conclusions.

## Purpose

The duodenal switch was proposed in 1987 by DeMeester et al. [1] as an alternative to Roux-en-Y gastric bypass for the treatment of bile reflux. The DeMeester’s intuition was adapted to bariatric surgery in 1989 by Hess and Marceau [2, 3], creating the biliopancreatic diversion procedure with duodenal switch (BPD-DS), to avoid the frequent marginal ulcers occurring following Scopinaro’s biliopacreatic diversion [4].

Long-term studies have demonstrated the BPD-DS to provide significantly greater weight loss than other bariatric procedures with concurrent sustained improvement in metabolic health [5]. However, BPD-DS should be considered a technically demanding operation. Complications are infrequent, but if a suture leak, a post-operative bleeding or an intestinal obstruction occur, consequences might be severe, ranging from hospital stay prolongation to sepsis, peritonitis, reoperations, or even death.

Laparoscopy has become the gold standard approach for virtually all bariatric surgeries since it was first used for gastric bypass by Wittgrove et al. 1993, to reduce the invasiveness of bariatric procedures [6]. With this purpose, and trying to simplify the technique used to perform the BPD-DS, Sánchez-Pernaute and Torres and coworkers introduced a new operation in the clinical practice in 2007: the “Single Anastomosis Duodeno-Ileal Bypass with Sleeve Gastrectomy” or SADI-S. Importantly, the SADI-S is based on BPD-DS, but it requires only one anastomosis [7]. Reducing the number of intestinal anastomoses should have some advantages over previous procedures, such as a lower probability of post-operative leaks or anastomosis strictures and obstructive problems related to internal herniation, a shortened operative time, and consequently less anesthetic-derived complications [8]. After initial interesting results [7], the technique became popular all over the world [9]. Single anastomosis duodeno-ileal bypass can be used as revisional surgery after failed sleeve gastrectomy (SADI) or after failed adjustable gastric banding and gastric bypasses [10, 11], but also as planned second-stage surgery (after sleeve gastrectomy) [12]. Based on retrospective studies, SADI-S is effective in achieving good initial weight loss and weight maintenance as well, with acceptable long-term “nutritional complications” [9]. In their 2016 position paper, the American Society for Metabolic and Bariatric Surgery (ASMBS) determined that SADI-S should be considered an “investigational” procedure, as they reckoned insufficient the existing randomized or prospective comparative data to draw any definitive conclusions regarding the safety, efficacy, and durability of this procedure compared with the standard BPD-DS [13]. In their updated statement (2020), the ASMBS endorsed the SADI-S as an appropriate metabolic procedure; however, they reported a lack of evidence regarding intestinal adaptation, nutritional issues, optimal limb lengths, and long-term weight loss/regain after this procedure. Therefore, they recommended caution in adopting this procedure, with attention to ASMBS-published guidelines on nutritional and metabolic support for bariatric patients, particularly for those who underwent malabsorptive procedures [14]. The International Federation for the Surgery of Obesity and Metabolic Disorders (IFSO) 2018 position statement also required more evidence to be obtained; but they acknowledged that the guidance for emerging procedure is the responsibility of the organizations, such as IFSO [15]. Considering the results of the systematic reviews in the 2018 position statement, the IFSO considered that the available short-term data demonstrated that this procedure satisfied both safety and efficacy concerns. However, they noted that data were insufficient to confirm long-term consequences of the operation. Hence, the IFSO did not reckon appropriate to continue to call the SADI-S “investigational” but recommended that long-term follow-up be continued. The IFSO updated statement (2021) [4] emphasized that SADI-S can help an obese person in achieving and maintaining significant weight loss with an improvement in metabolic health; however, nutritional deficiencies are emerging as long-term safety concern for SADI-S, and patients undergoing this procedure must be aware of this and counseled to stay in long-term multidisciplinary care. Moreover, the IFSO supports SADIS as a recognized bariatric/metabolic procedure, but highly encourages national and international registry and randomized controlled trials in the near future [4].

Therefore, the aim of this retrospective study is to analyze our monocentric experience, that is the first in Italy, on patients undergoing SADI-S, so to support international societies in the biannual evaluation, and to encourage national and international registries to obtain a better and more homogeneous data analysis.

## Methods

We conducted a retrospective review of prospectively collected data into a de-identified dedicated bariatric database of patients who underwent bariatric surgery (primary and revisional), between July 2016 and August 2021 at our tertiary referral center for bariatric surgery, Center of Excellence of the SICOb (Italian Society of Bariatric Surgery).

### Study endpoints

The primary endpoint was to evaluate the safety of the procedure in terms of 30th-day complication rate. The secondary endpoint was to evaluate the safety of the procedure in terms of mid-term complications.

### Patient population

All the patients who underwent SADI-S between July 2016 and August 2021 were identified among 2313 patients who underwent bariatric surgical procedures.

Continuous variables were expressed as median (interquartile range: Q1–Q3). Dichotomic variables were expressed as number and percentage (%).

For this study, follow-up was closed on the 31st January 2022.

The patients included in this study met the consensus criteria for bariatric surgery, fulfilled the national guidelines of SICOb [https://www.sicob.org/00_materiali/linee_guida_2016.pdf], and underwent primary (SADI-S) or revisional procedure (SADI) by laparoscopic or robotic approach. The patients were fully informed of the surgical technique, anesthesia, effects, and complications.

### Patients’ selection for SADI-S

In our clinical practice, candidates for SADI-S as a primary procedure are super-obese patients (BMI ≥ 50 kg/m^2^), especially with binge-eating habit and metabolic patients (e.g., diabetes mellitus type 2).

#### Revisional procedures

- Two-step’s procedure in young patients (age < 40 years) and/or challenging/complex cases (a combination of these features: man, BMI ≥ 60 kg/m^2^, previously major abdominal surgery)
- Inadequate weight loss and/or weight regain after sleeve gastrectomy and in other selected cases (previously laparoscopic adjustable gastric-banding, Roux-en-Y gastric bypass, one-anastomosis gastric bypass)
  Absolute contraindications are:
  - Barrett’s esophagus
  - Severe gastro-esophageal reflux disease
  - Major (> 4 cm) hiatal hernia

Indications for robotic approach: we used the robotic approach for “challenging cases”. “Challenging cases” are clinical features not evaluable by a single parameter;. indeed, it is determined by the occurrence of one or more of these conditions: patients with BMI ≥ 55 kg/m^2^, especially if males and/or with previously major abdominal surgery.

### Surgical techniques

All the procedures were performed by the same expert bariatric surgeon.

#### Laparoscopic single anastomosis duodeno-ileal bypass with sleeve gastrectomy

The patient lies in supine position, legs open. A 5-mm optical trocar is inserted in the left flank along the mid-clavicular line and pneumoperitoneum (14 mmHg) is made. Under visual control, one 12-mm trocar in the upper umbilical region and two 5-mm trocars, respectively, in the epigastrium and right hypochondrium, are placed. The procedure begins with the dissection of the gastrocolic ligament and proceeds with the preparation of the greater curvature, which is carried out cranially until the left diaphragm pillar is exposed and caudally to the pylorus. Then preparation of the first part of the duodenum is performed, until gastroduodenal artery is exposed. The next step is the vertical gastric resection, which is performed using a laparoscopic linear stapler and sized upon a 40 F orogastric bougie. At this point, the first part of the duodenum is sectioned, approximately 2 cm after the pylorus, using a linear stapler. Next caecum and last ileal loop are identified and 300 cm from ileocecal valve are counted: this is the point where gastro-ileal anastomosis will be made. The intestinal measurement is performed after infusion of 20 mg of Buscopan®, to get the maximum possible relaxation of the smooth muscle and perform the most accurate calculation of the common limb’s length. The selected loop is then anchored to the proximal sectioned duodenum with a PDS 3.0 stitch. At this point, a double layer manual termino-lateral antecolic duodeno-ileal anastomosis between the sectioned proximal duodenum and the previously identified ileal loop is made, using PDS 3.0 for the external anterior layer and Stratafix 2.0 for the external posterior and the internal layer. At the end of the reconstructive phase integrity of the anastomosis is verified with a blue methylene and pneumatic test. Sectioned stomach is then extracted through the left flank trocar site and sent for histological examination. Hemostasis is verified and a 19 F drainage is placed behind the anastomosis trough the left flank trocar site. Finally, fascial and skin closure is performed.

#### Robotic single anastomosis duodeno-ileal bypass with sleeve gastrectomy

The patient lies in supine position, legs open. A 12-mm optical trocar is inserted in the supraumbilical region and pneumoperitoneum (14 mmHg) is made. Under visual control, other 8-mm robotic trocars are placed along a horizontal line cranially to the 12-mm trocar, in the right and left hypochondrium and the right and left paramedian region. Then the caecum, the last ileal loop and the ileal loop where anastomosis will be made (at 300 cm from ileocecal valve) are identified. The selected loop is then inked and anchored to the omentum with a Vicryl 3.0 stitch. At this point, robotic docking is performed and the procedure follows the same above-mentioned steps.

#### Laparoscopic/robotic single anastomosis duodeno-ileal bypass (revisional procedures)

These procedures have the same steps described above. Obviously, gastric resection is not carried out because it is already performed, so surgery starts directly with the preparation of the duodenum.

### Post-operative protocol

A standard post-operative protocol personalized for bariatric patients was used [16]. The severity of post-operative complications was rated according to the Clavien-Dindo classification [17]. Routine follow-up with blood test analysis and physical examination were performed according to the SICOb guidelines [https://www.sicob.org/00_materiali/linee_guida_2016.pdf]. At discharge, the patients were advised to follow a strict dietary regimen which consists in 3 progressive phases (liquid, semisolid, and solid diet), each one lasting at least 2–3 weeks, with proteic, vitaminic, and mineral supplementations. Proteic supplementation (Protifar, Nutricia, Milan, Italy, 55 g per day during the first dietary phase and 15 g per day during the second one) is indicated because clinical practice guidelines for perioperative support of bariatric patients by the Endocrine and Obesity Societies recommend a daily intake of protein from a minimum of 60 up to 1.5 g/kg ideal body weight [18]. All the patients received FitForMe WLS Maximum® as vitamins and mineral supplementation. FitForMe WLS Maximum® is a customized multivitamin supplement for bariatric patients who underwent malabsorptive/hypoabsorptive procedures and contains elevated doses of multiple vitamins and minerals (see https://fitforme.it/product/pacchetto-wls-maximum/#1586345186042-d5da1ce5-60ed for details of composition). FitForMe WLS Maximum® is dosed as one capsule per day. All the patients received enoxaparin (4000 UI/0.4 mL) for 4 weeks and proton-pump inhibitor (PPI) (esomeprazole, 40 mg daily) for at least 6 months, as part of the standard post-operative protocol.

### Discharge criteria

The discharge is scheduled 24 h after the surgical procedure only if the following are conditions satisfied: no clinical complications or post-operative biochemical and imaging alterations occurred; oral alimentation is tolerated; autonomy in life activities is acquired; and the discharge is accepted by the patient.

### Statistical analysis

Basic demographic and clinical data were collected through reviews of patient charts and electronic databases.

Normal distribution was assessed with the Shapiro–Wilk test. Differences in median values were analyzed with *t*-test for repeated measures in parametric variables and Friedman’s test for non-parametric. The results are reported with a 95% confidence intervals.

Statistical analysis was conducted with SPSS 22.0 software for Windows (SPSS Inc., Chicago, IL).

This study was approved by our ethical board.

With the aim of reducing the errors in the interpretation of acronyms (SADIS, SADI), during the discussion we will use the term “SADI-S” to identify both primary procedures and revisional ones.

## Results

During the study period, over 2313 bariatric procedures were performed (2078 primary procedures and 235 revisional procedures). A total of 121 patients were scheduled for single anastomosis duodeno-ileal bypass with sleeve gastrectomy: 87 (71.9%) for primary procedure and 34 (29.1%) for the revisional one. The population characteristics are showed in Tables 1 and 2. Laparoscopic approach was performed in 99 (81.8%) cases; instead robotic approach was performed in 22 (18.1%) cases: we reported 66 laparoscopic SADIS, 33 laparoscopic SADI, 21 robotic SADIS and one robotic SADI. Overall, the median operative time was 120 (100–155) min. The median operative time for laparoscopic procedure was 120.0 (90–140) minutes, while the median operative time for robotic approach was 191.54 (165–205). The median docking time for robotic procedure was 10.5 (5–13) min. In this series, no other intra-abdominal procedures (such as cholecystectomy) have been performed, and no intraoperative complications were reported. No intraoperative leaks were detected at the methylene blue test. No intraoperative deaths occurred. No conversions were required, either from laparoscopic to open surgery, or from robotic to laparoscopic or open surgery. No 30th-day mortality was registered.

**Table 1:** Characteristics of study’s population

Patients	121
Age (years)	44.0 (39.0–51.0)
Height (cm)	168.0 (160.0–173.5)
Weight (kg)	142.0 (125.5–162.5)
BMI (kg/m^2^)	52.30 (48.75–57.05)
Male/female	41 (33.9%) / 80 (66.1%)
Smoking	
No	79 (65.3%)
Previously (≥ 6 months)	24 (19.8%)
Previously (< 6 months)	18 (14.9%)
Comorbidities (yes/no)	73 (60.3%) / 48 (39.7%)
HBP (yes/no)	46 (38%) / 75 (62%)
OSAS (yes/no)	39 (32.2%) / 82 (67.9%)
Diabetes	
No	92 (76.0%)
IGT	15 (12.4%)
DMT2	14 (11.6%)
Previous non-bariatric abdominal surgery	
No	59 (48.8%)
Laparoscopic	19 (15.7%)
Open	43 (35.5%)
Previous bariatric procedures	
No	80 (66.1%)
Sleeve gastrectomy	34 (28.2%)
Gastric banding	6 (4.9%)
Functional gastric bypass with an adjustable gastric banding	1 (0.8%)
BMI (kg/m^2^) at the first bariatric procedure	51.3 (44.0–57.475)
Procedure	
SADIS	87 (71.9%)
SADI	34 (29.1%)
Operative time (minutes)	120 (100–155)
Surgical approach	
Laparoscopic	99 (81.8%)
Robotic	22 (18.2%)
Intensive care unit (yes/no)	2 (1.7%) / 119 (98.3%)
Post-operative hospital stay (days)	2 (2–4)
Post-operative 30th-day complications (yes/no)	4 (3.3%) / 117 (96.7%)
Reoperation (yes/no)	2 (1.7%) / 119 (98.3%)
Pneumonia (yes/no)	2 (1.7%) / 119 (98.3%)
Bleeding (yes/no)	1 (0.8%) / 119 (99.2%)
Acute pancreatitis (yes/no)	1 (0.8%) / 119 (99.2%)
Trocar site hernia (yes)	1 (0.8%) / 119 (99.2%)
Other post-operative 30th-day complications: DVT. PE. AF. AMI. Sleeve leakage. Duodenal stump leakage. Duodeno-ileal anastomosis leakage. Small bowel perforation. Sleeve stenosis. Duodeno-ileal anastomosis stenosis. Small bowel twisting. Internal hernia	-
30th-day Hospital readmissions (yes/no)	1 (0.8%) / 119 (99.2%)
Late complications (yes/no)	4 (3.3%) / 117 (96.7%)
Incisional hernia (yes/no)	1 (0.8%) / 119 (99.2%)
Chronic diarrhea (yes/no)	3 (2.5%) / 118 (97.5%)
Malnutrition (yes/no)	2 (1.7%) / 119 (98.3%)
Other late complications: sleeve stenosis. Duodeno-ileal anastomosis stenosis. Small bowel twisting. Internal hernia. Duodeno-ileal anastomosis ulcer. Bile reflux	-
Other events (yes/no)	
Wernike-Korsakoff’s syndrome	1 (0.8%) / 119 (99.2%)
Toxic megacolon	1 (0.8%) / 119 (99.2%)
Follow-up time (months)	31 (14–39)
BMI	29 (25.1–35.65)
%EWL	79.8 (55.15–91.45)
DBM	2 (2–3)
%TWL	57.0650 (43.3925–71.3475)

*BMI* body mass index *HBP* high blood pressure *OSAS* obstructive sleep apnea syndrome *IGT* impaired glucose tolerance *T2DM* type 2 diabetes mellitus *DVT* deep vein thrombosis *PE* pulmonary embolism *AF* atrial fibrillation *AMI* acute myocardial infraction *SADIS* single anastomosis duodeno-ileal bypass with sleeve gastrectomy *SADI* single anastomosis duodeno-ileal bypass in previously sleeve gastrectomy *%EWL* percentage of excess weight loss *DBM* day bowel movements *%TWL* total weight loss

**Table 2:** Characteristics of study’s population according to the procedures (SADI-S and SADI) performed

	SADIS	SADI
Patients	87 (71.9%)	34 (28.1%)
Age (years)	45.0 (40.0–52.0)	41.0 (36.5–50.5)
Height (cm)	168.0 (153.0–175.0)	168.0 (158.0–170.5)
Weight (kg)	152.0 (135.0–171.0)	115.5 (106.5–138.5)
BMI (kg/m^2^)	53.600 (51.400–59.100)	52.950 (39.375–49.250)
Male/female	33 (37.9%) / 54 (62.1%)	8 (23.5%) / 26 (76.5%)
Smoking		
No	53 (60.9%)	26 (74.5%)
Previously (≥ 6 months)	21 (24.1%)	3 (10.8%)
Previously (< 6 months)	13 (15.0%)	5 (14.7%)
Comorbidities (yes/no)	58 (66.7%) / 29 (33.3%)	15 (44.1%) / 19 (55.9%)
HBP (yes/no)	35 (40.2%) / 52 (59.8%)	11 (32.5%) / 23 (67.5%)
OSAS (yes/no)	33 (37.9%) / 54 (62.1%)	6 (17.4%) / 28 (82.6%)
Diabetes		
No	63 (72.4%)	29 (85.2%)
IGT	13 (14.9%)	2 (7.4%)
DMT2	11 (12.7%)	2 (7.4%)
Previously non-bariatric abdominal surgery		
No	49 (56.3%)	10 (29.4%)
Laparoscopic	10 (11.5%)	9 (26.5%)
Open	28 (32.2%)	15 (44.1%)
Previously bariatric procedure		
No	80 (92.0%)	0
Sleeve gastrectomy	0 (0%)	34 (100%)
Gastric bending	6 (7.2%)	0 (0%)
Vertical gastroplastic	0 (0%)	0 (0%)
Functional gastric bypass with an adjustable gastric banding	1 (0.8%)	0 (0%)
Operative time (minutes)	133 (114–165)	91 (65–120)
Intensive care unit (yes/no)	2 (2.3%) / 85 (87.7%)	0 (0%) / 34 (100%)
Postoperative hospital stay (days)	2 (2–4)	2 (2–4)

*SADIS* single anastomosis duodeno-ileal bypass with sleeve gastrectomy, *SADI* single anastomosis duodeno-ileal bypass in previously sleeve gastrectomy, *BMI* body mass index *HBP* high blood pressure *OSAS* obstructive sleep apnea syndrome, *IGT* impaired glucose tolerance, *T2DM* type 2 diabetes mellitus

We reported 30th-day post-operative complications in 4 (3.3%) patients, and 2 of them required surgical treatment.

The first case was a 51-year-old man with a preoperative BMI 52.3 kg/m^2^ who underwent laparoscopic SADIS. Right after surgery, he developed severe acute necrotizing pancreatitis of unknown origin (not biliary). Further surgery with debridement and necrosectomy was necessary (Clavien-Dindo IIIb): during this procedure, due to the severity of the abdominal situation, an anastomotic breakdown was required, and gastrostomy and nutritional jejunostomy were made. Pancreatitis was then treated with open abdomen technique and vacuum-assisted therapy with regular surgical revisions. The patient required intensive care support (Clavien-Dindo IV) for almost all the hospital stay. Gradually, we observed a clinical improvement which allowed to restore the intestinal continuity, at 4 months from first surgery, performing a one anastomosis gastric bypass (CASE 1).

The second case was a 42-year-old woman with preoperative BMI 54.6 kg/m^2^, who underwent robotic SADIS. On POD2, the patient developed abdominal pain located on the superior umbilical trocar site and bilious vomiting after upper gastrointestinal contrast study with water soluble contrast. On physical examination, a swelling was noted on the trocar site, suspicious to be an incarcerated hernia with subileus. So urgent surgical revision (Clavien-Dindo IIIb) was mandatory, with reduction of the herniated intestinal loop and fascial closure (CASE 2).

The third case was a 62-year-old woman with a preoperative BMI 51.4 kg/m^2^ who underwent robotic SADIS. The patient developed fever on POD 2. Blood tests showed elevated acute phase reactants of inflammation. Despite negative upper gastrointestinal contrast study, the persistent fever demanded a CT scan with intravenous and oral contrast, which showed pneumonia. The patient had intravenous antimicrobial therapy (Clavien-Dindo II), following the infectious disease consultant’s indications, and her clinical condition improved rapidly (CASE 3).

The fourth case was a 57-year-old woman with preoperative BMI 57.0 kg/m^2^ who underwent robotic SADIS and was discharged on POD 2. She had uneventful course for 2 weeks after surgery, when she developed fever with elevated acute phase reactants of inflammation. An abdominal CT scan with intravenous and oral contrast was mandatory and showed a 5-cm-hematic collection near to the stomach suture line, without evidence of fistula. Consequently, the patient was readmitted and put on empirical intravenous antibiotic therapy (Clavien-Dindo II). Rapid and complete normalization of body temperature was observed (CASE 4).

Four patients (3.3%) developed late complications.

The first patient was a 19-year-old man with preoperative BMI 58.1 kg/m^2^ who underwent laparoscopic SADIS. He had no compliance to the dietetic regimen and to the vitaminic and oligoelement supplementation, so in the 6 following months, he developed a severe malnutrition state and a Wernicke Korsakoff syndrome. After correction of the nutritional deficits with parenteral nutrition, owing to the low compliance of the patient, we decided to turn the initial procedure into a Roux-en-Y gastric bypass (Clavien-Dindo IIIb) (CASE 5).

The second and third patients were a man and a woman, 50 and 48 years old, respectively, with preoperative BMI 43.3 and 46 kg/m^2^, who underwent laparoscopic SADI. One year after the initial surgery, they developed chronic diarrhea unrelated to *Clostridium difficile*; he recovered with antibiotic therapy per os and pre/probiotic supplementation (Clavien-Dindo II) (CASE 6). The third patient’s conditions were worse, with electrolyte imbalance and initial malnutrition due to rapid weight loss, but the patient was not willing to refer to the reference bariatric center. So, an intravenous correction of the electrolyte imbalance was necessary (Clavien-Dindo II) (CASE 7).

The fourth patient was a 48-year-old woman with preoperative BMI 51.8 kg/m^2^, who underwent laparoscopic SADIS. At 2 years from surgery, she presented rectorrhagia, and new onset ulcerative colitis was diagnosed. Fast progression of the disease determined a malnutrition state with electrolyte imbalance that required prompt corrections. She developed also a colic perforation related to toxic megacolon, which required urgent total colectomy with end ileostomy (Clavien-Dindo IV) (CASE 8).

At a median follow-up time of 31 (14–39) months, the median TWL (total weight loss) was 57.0650 (43.3925–71.3475)%; the median %EWL (percentage of excess weight loss) was 79.8 (55.15–91.45); the median BMI was 39 (25.1–36.65) kg/m^2^, and the median daily bowel movements was 2 (2–3).

In Tables 3 and 4, the baseline characteristics and bariatric and metabolic results in a subpopulation (66 patients) that completed 2 years of follow-up are reported. We excluded 2 patients (CASES 1 and 5) due to their clinical history.

**Table 3:** Characteristics and bariatric and metabolic outcomes of patients that completed 2-year follow-up

Patients	66
Age (years)	42.5 (38.0–50–2)
Height (cm)	168.00 (160.75–172.75)
Weight (kg)	143.50 (129.00–172.75)
BMI (kg/m^2^)	53.050 (48.793–58.318)
Male/female	19 (28.8%) / 47 (71.2%)
Smoking	
No	33 (50.0%)
Previously (≥ 6 months)	17 (25.8%)
Previously (< 6 months)	16 (24.2%)
Comorbidities (yes/no)	48 (72.7%)
HBP (yes/no)	31 (47.0%) / 35 (53.0%)
OSAS (yes/no)	30 (45.5%) / 36 (54.5%)
Diabetes	
No	50 (75.8%)
IGT	6 (9.1%)
DMT2	10 (15.2%)
Previous bariatric procedures	
No	48 (72.7%)
Sleeve gastrectomy	14 (21.2%)
Gastric banding	4 (6.1%)
Functional gastric bypass with an adjustable gastric banding	0 (0%)
BMI (kg/m^2^) at the first bariatric procedure	
Sleeve gastrectomy	50.70 (42.44–54.06)
Gastric banding	54.58 (50.11–59.83)
Sleeve gastrectomy	40.00 (36.00–81.00)
Gastric banding	126.00 (87.75–219–00)
Procedure	
SADIS	52 (79.8%)
SADI	14 (21.2%)
BMI (kg/m^2^) at 2 years	27.3 (23.1–31.4)
%EWL at 2 years	85.3 (72.1–96.0)
DBM at 2 years	2 (2–3)
%TWL at 2 years	75 (49–100)
HBP at 2 years	
Partial resolution	1
Complete resolution	13
OSAS	
Partial resolution	2
Complete resolution	25
Diabetes at 2 years	
Partial resolution	5
Complete resolution	10

*BMI* body mass index, *HBP* high blood pressure, *OSAS* obstructive sleep apnea syndrome, *IGT* impaired glucose tolerance, *T2DM* type 2 diabetes mellitus, *SADIS* single anastomosis duodeno-ileal bypass with sleeve gastrectomy, *SADI* single anastomosis duodeno-ileal bypass in previously sleeve gastrectomy, *%EWL* percentage of excess weight loss, *DBM* day bowel movements, *%TWL* total weight loss

**Table 4:** Evolution of the nutritional markers of the patients that completed 2-year follow-up

	T0	T1	*p*
Hemoglobin (g/dL)	13.3 (12.15–13.75)	11.9 (11.4–13.2)	0.012
Total serum protein (g/L)	78.0 (74.5–81.5)	66.0 (63.5–70.0)	0.088
Albumin (g/L)	41 (39–43)	38 (34.5–42)	0.078
Calcium (mg/dL)	9.6 (9.2–9.8)	8.900 (8.650–9.205)	< 0.001
Sodium (mmol/L)	140 (139–141)	141 (139–141)	0.471
Potassium (mmol/L)	3.90 (3.60–4.05)	4.00 (3.75–4.40)	0.241
Triglycerides (mg/dL)	109.00 (79.50–190.50)	69.50 (61.25–81–25)	0.059
Glycated hemoglobin (mmol/mol)	46.0 (43.0–46.0)	30.0 (44.5–52.5)	0.176
Parathyroid hormone (pg/mL)	83.3 (48.2–100.4)	57.550 (34.025–111.000)	0.930

*T0* baseline, *T1* 24 months

## Discussion

The SADI-S was described [7] and subsequently routinely introduced in the clinical practice [19] by Sánchez-Pernaute and Torres and coworkers in 2007. The technique required the elimination of Roux-en-Y reconstruction of the original BPD-DS to perform a Billroth II-type one-loop duodeno-ileal anastomosis instead [7]: if the pylorus is preserved, a Roux-en-Y reconstruction should not be required, as the pancreatic and biliary juices face a natural barrier protecting the stomach. The main question in a one-loop reconstruction was to guess the adequate length of the common channel. At the beginning, Sánchez-Pernaute and Torres performed a common limb of 200 cm [7]. After the initial experience [19] and the increased SADI-S popularity all over the world, the length of the common channel has been moved to 250/300 cm, since longer common limbs (300 cm) are associated with fewer long-term nutritional and malabsorptive complications [20]. Eliminating one anastomosis should yield a reduction in operative time, anesthesia time and post-operative complications probability [19]. To date, initial long-term results become available [20], and this is a particularly interesting topic for the international organizations such as ASMBS and IFSO [14, 15]. These data suggest that SADI-S offers good results for the treatment of both morbid obesity and its co-existing conditions [20, 21].

In this study, we present the results of our SADI-S’ experiences in an Italian high volume bariatric center. We performed the first SADIS case in July 2016, and it was robotic; the first laparoscopic SADIS and the first laparoscopic SADI were performed in February 2017, and the only robotic SADI was performed in March 2021. Overall, the outcomes of 121 patients were reported. To our knowledge, this is the first monocentric Italian study on this procedure.

In our experience, SADI-S is a safe procedure with low post-operative complications. In the present study the starting median BMI was 52.3 kg/m^2^; the median follow-up time was 31 months; and the median %EWL was 79.8 and the median %TWL was 57.0650. These data and the other preoperative demographic characteristics are in line with the existing evidence [9, 19, 20, 22]. We registered a median operative of 120 min. Other authors reported lesser results [20]; however, these data are not always available in other studies [4, 9, 19]. However, our series is heterogeneous for the surgical approach (laparoscopic vs robotic) and for the type of procedure (primary versus revisional), and this may influence the interpretations of our results. Nevertheless, another important aspect which influences the operative time is the learning curve effect. As we reported in another study [23], we always considered the number of the laparoscopic and robotic bariatric procedures that has been performed by the operator(s). In this context, all the SADI-S were performed by the same surgeon (M. R.), who performed more than 1000 laparoscopic bariatric procedures and more than 30 robotic procedures prior to perform the first robotic SADI-S, and in all cases the duodeno-ileal anastomosis was handsewn in double layer. Indeed, the study of Surve et al. [20] reported a mean operative time of 67.2 min for 750 procedures, performed by 3 surgeons. Anyway, we must underline that both in laparoscopic procedures and in robotic ones, the duodeno-ileal anastomosis was manual (hand-sewn), and this may partly explain the major operative times compared to others’ experiences [9]. Moreover, we experienced a longer median operative time for robotic approach (191.54 vs 120.00 min). Indeed, these unpublished data were reported as oral communication at the 29th EAES Congress (Barcellona, Spain, 24–27 Novembre 2021). In our communications, we analyzed a propensity score matching case control series (robotic vs laparoscopic approach for SADI-S) in super-obese patients, where we indicated a longer operative time in robotic groups (191.54 vs 130.00 min, < 0.001), but comparable post-operative complications (3 vs 0 cases, *p* = 0.233) and shorter median post-operative stay (2 vs 3 days, *p* = 0.062). The median operative times for robotic approach in our experience is shorter than other anecdotal experiences: Tat et al. [24] reported an average median of 204 min on 11 case experiences; Tarascó Palomares et al. [25] reported a single-case experience with a total operative time of 240 min (of which 165 min were required to complete the SADIS procedure and 75 min to reduce and repair an umbilical hernia); and Vilallonga et al. [26] experienced an average operative time of 145 min in 3 robotic SADI cases. Our data underlined the advantage of the robotic approach to perform these procedures in super-obese patients. In fact, conventional laparoscopy comes with some technical limitations, which are amplified by the difficulties that accompany obese patients in general and super-obese patients in particular. In terms of surgical challenges, these limitations include space constraints (often caused by increased liver size), intra-abdominal fat and a thick abdominal wall, which further restrains instrument handling typical used in minimally-invasive surgery [5, 27]. Moreover, current literature shows that the overall complication rate of laparoscopic bariatric procedures is as great as 20%, and leak rates may reach up to 5.1% [28, 29]. As a result, the patients experience reoperations, prolonged hospital stays, and possible serious life threats. Thus, it is evident that improvements in clinical outcomes driven by advanced technologies in bariatric surgery are needed, especially in this special population [30]. The advantages of using the robotic system include greater dexterity and precision in tissue manipulation, especially in challenging cases and in anatomical regions that are difficult to access: this may result in fewer conversion rates [31] and probably fewer short-term complications [32]. SADI-S is a malabsorptive/hypoabsorptive challenging multi-quadrant procedure and is mostly indicated in the treatment of “complex” bariatric patients (BMI ≥ 50 kg/m^2^, metabolic patients and revisional surgery), so we trust that it is the ideal procedure to benefit from robotic technologies.

We registered a 3.3% of early (30th day) post-operative complications, with 0.8% readmission rate and 1.7% reoperation rate, substantially similar to the studies of Surve et al. [20] and Vilallonga et al. [33]. We did not experience anastomosis, sleeve, or duodenal-stump leakages, when anastomotic leakage is one of the most serious complications after bariatric surgery [34, 35]. Torres et al. [19] reported 4% (2 cases/50 patients) anastomotic leakage in the early experience, while Surve et al. [9] experienced 9 cases of this complication over 1328 patients (0.6%). Vilallonga et al. [33] reported in a recent review on 398 patients a complication rate of 4.1% with an incidence of 1.9% of leakage. The incidence rate of anastomosis complication after SADI-S is low compared with the reported rate of anastomotic complication after Roux-en-Y gastric bypass and BPD-DS. In both these procedures, anastomotic leak can occur at the gastro-jejunal and jejuno-jejunal anastomoses. For instance, the most frequently reported leak location after Roux-en-Y gastric bypass is at the gastro-jejunal anastomosis (68%), although some have reported greater mortality from jejuno-jejunal anastomosis’ leak [36]. The reported incidence of leakage varies from 0.1 to 5.6% [37]. Clearly SADI-S requires only one anastomosis compared with 2 of the Roux-en-Y gastric bypass and BPD-DS: this is another of its advantages. The reported incidence, instead, of anastomotic leakage after BPD-DS varies from 0.5 to 6% [38, 39]. In our series, we did not register any marginal ulcer (anastomotic side ulcer), which is a well-known complication of gastro-jejunal anastomosis, with approximate incidence from 0.6 to 20% and unclear etiology [40]. The possible contributing factors include local ischemia, anastomotic tension, increased gastric acidity, tobacco use, non-steroidal anti-inflammatory drug use and chronic irritation caused by the suture materials [11, 41]. Ulcer is a consistent finding in Roux-en-Y reconstruction, so that Roux-en-Y reconstruction may be defined as ulcerogenic, with this being just an accepted outcome of the technique [9]. The reported incidence of the anastomotic ulcer after BPD-DS varies from 0.2 to 1.9% [42, 43]. Surve et al. [9] reported 0.1% of marginal ulcer, without routine use of proton-pomp inhibitor therapy post-operatively. The loop configuration in SADI-S procedure maintains contact between pancreatic enzymes, bile salts, and food: this is probably the reason for the reduction of the incidence of this complication. Furthermore, the routine use of proton-pomp inhibitor therapy post-operatively for a long period of time (at least 6 months) has contributed to our results. For the other early complications, our results are similar to those reported in other major experiences [9, 20].

The median follow-up time is shorter than other studies [9, 20], and since many medium- and long-term complications are influenced by the duration of the observation time, our data must be considered partially preliminary. We did not experience any internal hernias: similarly, other authors reported anecdotal rates [20, 44]. Obstruction related to internal hernia is one of the complications after Roux-en-Y gastric bypass and biliopancreatic diversion [45, 46]. In 1900, Peterson was the first surgeon who described internal hernia after gastro-jejunal anastomosis [9]. The reported incidence of internal hernias after Roux-en-Y varies from 0.5 to 16%[47, 48]. The possible locations for intestinal hernias include the opening of the transverse mesocolon, through which the Roux-en-Y limb is carried upwards to be connected to the gastric pouch (67%), the small bowel mesenteric defect at the jejuno-jejunal anastomosis (21%), and the space between the transverse mesocolon and Roux-en-Y limb mesentery (7.5%) [48]. Obeid et al. [49] reported long-term outcomes after Roux-en-Y gastric bypass with 10 to 13 years of follow-up. The incidence of internal hernia post-Roux-en-Y gastric bypass was 12.8% at an average of 3.7 years [49]. The reported incidence of internal hernias after BPD-DS varies from 0.4 to 18% [50, 51]. In both procedures, articles with long-term follow-up indicate internal hernias per year > 1%, regardless of the method used to close the internal hernia [9]. In SADI-S, the chances of internal hernias are lower as the mesentery is not closed but wide open. Some authors [20] believe that there will be some incidence of volvulus in the long term, but very few incidences of vascular compromise, as the space is large.

As previously reported, the median follow-up in our study is shorter than the median follow-up to date available in the newer studies [4, 20]: thus, our data may be partially affected by this. In all the procedures in our series, the length of common channel is 300 cm, established to reduce late complications, as reported by other authors [20]. With a common channel of 300 cm, a total of 3 (2.5%) patients experienced chronic diarrhea, coherently with the study of Surve et al. [20]. One patient (0.8%) in our series required conversion to Roux-en-Y gastric bypass to treat malnutritional status. Surve et al. [20] reported that 22 (2.9%) patients required common channel lengthening for chronic diarrhea and 2 (0.2%) patients for malnutrition, with a total of 5.3% of patients that required reoperation. Sethi et al. [51] reported 4.1% of reoperation due to severe malnutrition. Instead Sánchez-Pernaute et al. [52] reported in their study that 6 (6.1%) of patients required reoperation. We did not experience duodeno-ileal anastomotic stenosis, probably connected to its hand-sewn nature. Surve et al. [20] reported, instead, 5 (0.3%) cases of stricture, with 3 (2.4%) patients who had received stapler anastomosis [20]. Another theoretic concern is bile reflux, that is a potential late complication following a Billroth II-type reconstruction. This configuration, as currently performed worldwide, has a 1% incidence of bile reflux causing reintervention. In our series, we did not experience this complication, in line with that reported by Surve et al. [20] (0.1%). Likely, this is related to lower incidence of bile reflux due to the post-pyloric reconstruction of the single anastomosis, and probably if this complication occurs, it could be technique-related [20]. To support this theory, Surve et al. [53] reported a complication of retrograde filling of the afferent limb, causing symptoms like partial bowel obstruction caused by adhesion around the duodeno-ileal anastomosis and scar tissue from the gallbladder fossa after cholecystectomy to the afferent limb [53]. Last but not least, at median follow-up of 31 months, the results of median daily bowel movements, median %EWL, and median BMI are comparable to those reported by other series [9, 20]. Major %EWL is reported by some authors [19] who used a shorter common limb in their reconstructions.

We performed a subgroup analysis in patients that completed 2 years of follow-up. This subgroup had a median BMI of 27.3 kg/m^2^ and a median %EWL of 75. Our results are comparable to those reported in the review of Spinos et al. [54]. Similarly, the percentage of patients with comorbidities improvement or resolution are comparable with most of the series published [54].

This is the first national monocentric retrospective study for SADI-S; however, several limitations of our analysis should be noted. First of all, this a retrospective study over a long period time. Second, the median follow-up is too short to certainly define the complication rate at middle- and long-term. Moreover, the bariatric and metabolic results over the long term have not been reported; 54.5% of patients have completed a 2-year follow-up, with only few having reached the 5-year follow-up.

In conclusion, SADI-S is a safe procedure, thanks to its low rate of early complications. Our experience confirms the reproducibility of the technique, provided that it is performed in referral centers. So we can conclude that it can be one of the procedures of choice in the treatment of super-obese patient and in case of failed results after sleeve gastrectomy. However, we reckon necessary the establishment of national and international registries to obtain more homogeneous data and, most of all, to analyze the bariatric and metabolic effects over the long period in a sufficiently large number of patients, so to exclude a subpopulation bias effect.

## Data Availability

The datasets generated during and/or analyzed during the current study are available from the corresponding author on reasonable request.
